# The Spontaneously Diabetic Torii Rat: An Animal Model of Nonobese Type 2 Diabetes with Severe Diabetic Complications

**DOI:** 10.1155/2013/976209

**Published:** 2013-02-26

**Authors:** Tomohiko Sasase, Takeshi Ohta, Taku Masuyama, Norihide Yokoi, Akihiro Kakehashi, Masami Shinohara

**Affiliations:** ^1^Biological/Pharmacological Research Laboratories, Central Pharmaceutical Research Institute, Japan Tobacco Inc., Osaka 569-1125, Japan; ^2^Toxicology Research Laboratories, Central Pharmaceutical Research Institute, Japan Tobacco Inc., Kanagawa 257-0024, Japan; ^3^Division of Cellular and Molecular Medicine, Department of Physiology and Cell Biology, Kobe University Graduate School of Medicine, Kobe 650-0017, Japan; ^4^Division of Molecular and Metabolic Medicine, Department of Physiology and Cell Biology, Kobe University Graduate School of Medicine, Kobe 650-0017, Japan; ^5^Department of Ophthalmology, Saitama Medical Center, Jichi Medical University, Saitama 330-8503, Japan; ^6^Planning and Development Section, CLEA Japan Inc., Tokyo 153-8533, Japan

## Abstract

The Spontaneously Diabetic Torii (SDT) rat is an inbred strain of Sprague-Dawley rat and recently is established as a nonobese model of type 2 diabetes (T2D). Male SDT rats show high plasma glucose levels (over 700 mg/dL) by 20 weeks. Male SDT rats show pancreatic islet histopathology, including hemorrhage in pancreatic islets and inflammatory cell infiltration with fibroblasts. Prior to the onset of diabetes, glucose intolerance with hypoinsulinemia is also observed. As a result of chronic severe hyperglycemia, the SDT rats develop profound complications. In eyes, retinopathy, cataract, and neovascular glaucoma are observed. Proliferative retinopathy, especially, resulting from retinal neovascular vessels is a unique characteristic of this model. In kidney, mesangial proliferation and nodular lesion are observed. Both peripheral neuropathy such as decreased nerve conduction velocity and thermal hypoalgesia and autonomic neuropathy such as diabetic diarrhea and voiding dysfunction have been reported. Osteoporosis is another complication characterized in SDT rat. Decreased bone density and low-turnover bone lesions are observed. Taking advantage of these features, SDT rat has been used for evaluating antidiabetic drugs and drugs/gene therapy for diabetic complications. In conclusion, the SDT rat is potentially a useful T2D model for studies on pathogenesis and treatment of diabetic complications in humans.

## 1. Introduction

 In recent years, with economic development and social modernization, the number of diabetic patients has been increasing worldwide, including developing countries, posing a global problem in terms of human suffering as well as medical costs [1, 2]. The International Diabetes Federation (IDF) estimated the number of diabetic patients to be 366 million in 2011 [3], and that it will reach 552 million by 2030 unless effective measures are taken. 

 At present, complex interactions between genetic factors and environmental factors in the pathogenesis of diabetes are undergoing extensive study. However, the relationships between genetic and environmental factors are difficult to verify, and direct obtainment of information from humans has intrinsic limitations due to significant ethical restrictions. The use of experimental animal models is essential to resolve these problems, and results of basic research in animal models of diabetes may be useful to clarify the pathogenetic mechanism of human diabetes as well as the causes of complications and in the development of drugs for diabetes. In fact, many animal models of diabetes have contributed to clinical research of diabetes. It is important to develop animal models that correspond to various pathological conditions of human diabetes, and it is urgently necessary to develop models of diabetic complications that can reflect human diabetes, because the number of patients with type 2 diabetes (T2D) is rapidly increasing and progression of complications significantly affects the prognosis of diabetic patients.

**Figure 1:**
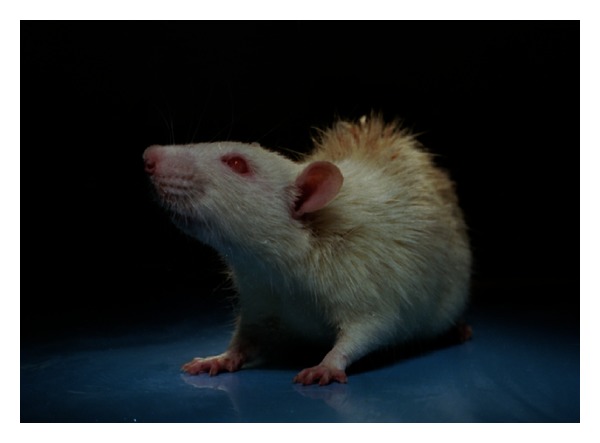
Appearance of 35-week-old male SDT rat [4].

Although there are many T2D model animals such as Goto-Kakizaki (GK) rats, Zucker Diabetic Fatty (ZDF) rats, and Otsuka Long-Evans Tokushima Fatty (OLETF) rats, and these animal models show diabetic complications, severe diabetic retinopathy has not been observed in the existing models. In these circumstances, Spontaneously Diabetic Torii (SDT) rat (Figure 1) has been established as a model of nonobese T2D with three major complications, including ocular complications [4–6]. This paper provides an overview of the findings from SDT rats such as pathology of diabetes.

## 2. Origin of Animals and Establishment of Inbred Strain

**Figure 2:**
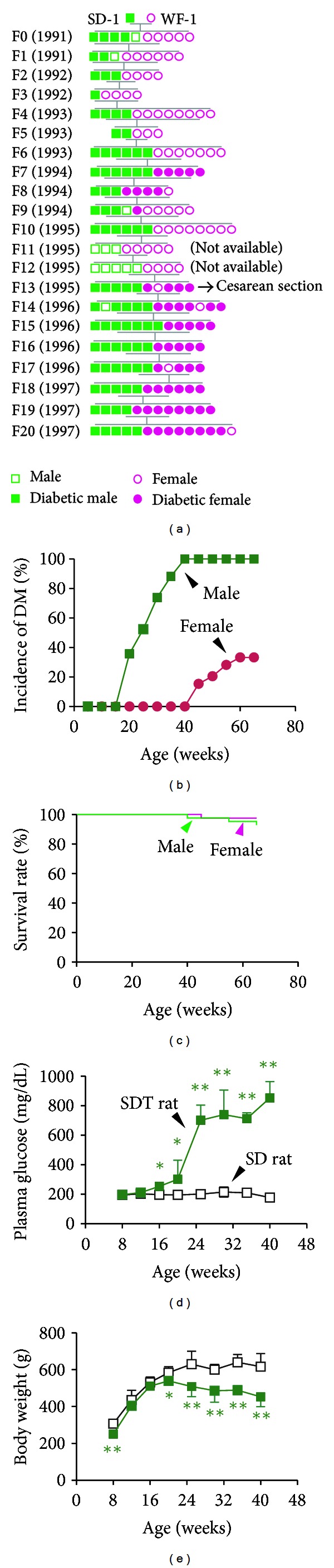
Pedigree maps and general profiles of SDT rats. Pedigree maps of nucleus line (a) and basic profiles of SDT rats. The cumulative incidence of DM was 100% at 40 weeks in male SDT rats (b) and the survival rates at 65 weeks was over 90% (c). The plasma glucose levels in male SDT rats reached 700 mg/dL by 25 weeks (d) and a decrease in body weight was observed from 20 weeks (e). Green square: male SDT rats, pink circle: female SDT rats. Open symbols represent male or female SD rats. Data represent means ± SD (*N* = 5–8). *P < 0.05, **P < 0.01 (versus age-matched SD rats, unpaired *t*-test). Figures are modified from [4, 6].

 In 1988, Shinohara found five nonobese diabetic rats with polydipsia, polyphagia, polyuria, and sugar urine among approximately 12-month-old elderly male Sprague-Dawley (SD) rats, which were bred at the laboratory of Torii Pharmaceutical Co., Ltd. (305 males and 306 females) after purchase from Charles River Laboratories Japan Inc. for long-term studies of spontaneous lesions. These animals were mated with young normal female rats of the same strain to successfully generate diabetic F1, and then attempts were made to preserve the diabetic trait in a closed colony (Figure 2(a)). In 1991, some animals in the diabetes-preserved colony developed diabetes at 4 to 5 months of age, leading to sib mating based on positive urine sugar in male rats. In 1997, a new inbred strain of nonobese T2D rats was established and named SDT rat [4–6]. In the process of strain breeding, the prevalence of diabetes in male rats was 90% or more in the F4 generation and 100% in the F9 and subsequent generations. Diabetes tended to occur earlier in later generations and occurred at approximately 4 months of age in the F7. This strain of rats was characterized by the development of diabetes only in males since its discovery, but the disease was sporadically observed in females aged 9 months or older in the F7 and subsequent generations [5]. Currently, SDT rat is distributed by CLEA Japan, Inc. (Tokyo, Japan) as SDT/Jcl rat.

## 3. Phenotype and Clinical Characteristics

### 3.1. Characteristics of Diabetes

 A clear sex difference is observed in the onset of diabetes in SDT rats (Figure 2(b)). While males developed diabetes at approximately 20 weeks of age with a cumulative incidence of 100% at 40 weeks, females developed it at 45 weeks with a cumulative incidence of as low as 33% at 65 weeks [6]. It is suggested that this sex difference may be partly attributed to estrogen, which inhibits the development of diabetes in females [7]. The survival rate at 65 weeks was 92% in males and 97% in females, showing that the rats survive hyperglycemia without insulin treatment (Figure 2(c)) [6]. The fasting and nonfasting blood glucose levels markedly increased at 20 weeks and thereafter, reaching 700 mg/dL or more at 30 weeks with polyuria characterized by severe sugar urine as well as polydipsia/polyphagia (Figure 2(d)). In SDT rats, development of hyperglycemia may be more dependent on decreased insulin secretion than insulin resistance, as shown by the fact that the blood insulin concentration tended to be lower than that in normal SD rats even before the onset of diabetes, and marked hypoinsulinemia developed after the onset of hyperglycemia [8, 9], indicating that this strain of rat is a model of nonobese T2D associated with impaired insulin secretion. Compared with normal SD rats, body weight and body-mass index (BMI) were similar before the onset of diabetes, but decreased with age after the onset (Figure 2(e)) [6, 8, 10].

### 3.2. Glucose Tolerance/Insulin (In Vivo and In Vitro)

**Figure 3:**
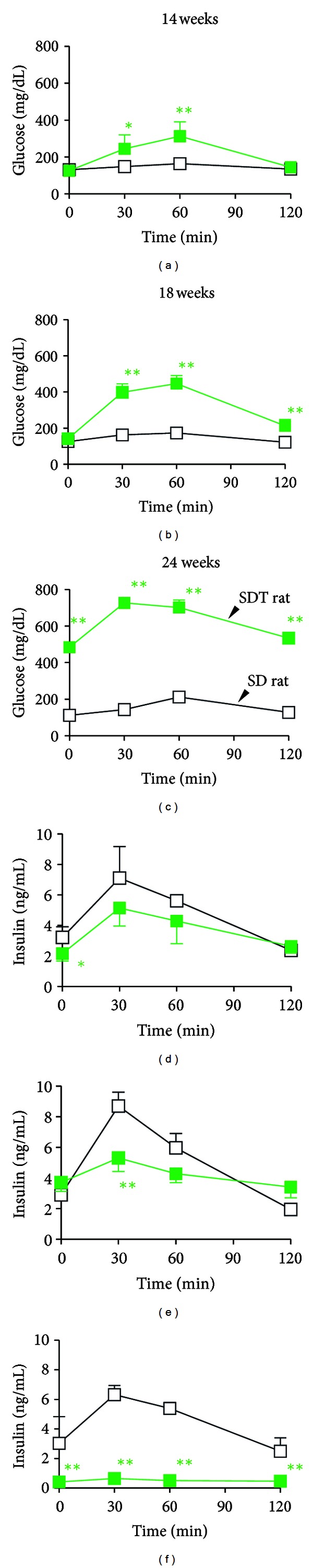
Oral glucose tolerance test (OGTT) in SDT rats. Changes in plasma glucose and insulin concentrations during OGTT (2 g/kg). At 14 and 18 weeks, SDT rats were nondiabetic but the marked elevation of plasma glucose and lower insulin concentration were observed after glucose loading. At 24 weeks, SDT rats were diabetic and showed further elevation of plasma glucose level because of diminished insulin secretion. White square: male SD rats, green square: male SDT rats. Data represent means ± SD (*N* = 6). *P < 0.05, **P < 0.01 (versus SD rats, unpaired *t*-test). Figures are modified from [8].

It is clinically known that glucose tolerance decreases before the onset of T2D. In oral glucose tolerance test (OGTT) in SDT rats, glucose tolerance markedly decreased at least 2 months before manifestation of hyperglycemia (around 14 weeks old), and the rate of rise in blood sugar level after glucose load increased with age. In male rats, the severity of impaired glucose tolerance before the onset of diabetes was closely correlated with the age at onset of disease. Impaired glucose tolerance was related to decreased insulin secretory response after glucose load, and decrease in the fasting plasma insulin concentration and loss of insulin secretory response after glucose load were observed after the onset of diabetes (Figure 3) [8, 11]. In addition, the insulin secretion level in pancreatic *β*-cells from SDT rats after glucose treatment markedly decreased at 12 weeks of age and thereafter compared with normal SD rats. Likewise, the mRNA expression levels for GLUT2 and glucokinase in the isolated pancreatic islets markedly decreased at 12 weeks and thereafter in SDT rats [12]. In female rats, glucose tolerance also decreased at 25 weeks and thereafter, but insulin was secreted after glucose load, indicating that some factors cause insulin resistance or insulin requirement in the females, unlike in the males [13]. It has also been reported that increased insulin secretion from hypertrophic pancreatic islets delayed the onset of hyperglycemia in high-fat diet-fed SDT rats [14]. 

### 3.3. Blood/Urine Biochemical Parameters and Lipids

 As for the biochemical parameters, urine protein, blood urea nitrogen (BUN), glycated hemoglobin (HbA1c), and triglycerides (TG) markedly increased with the development of hyperlipidemia at 35 weeks of age and thereafter [6]. In male rats, the blood TG concentration after fat load was high with normal TG absorption from the small intestine before the onset of diabetes, suggesting that the TG clearance is already impaired before the onset of disease. It is also suggested that not only the impairment of TG clearance, but also increased TG absorption from the small intestine occurs after the onset of disease. In addition, increased TG absorption may result from the physical increase in TG inflow associated with diabetes-related hyperphagia-induced hypertrophy of the small intestine as well as the increase in enzymes involving in TG absorption in the small intestine [15, 16]. Plasma ghrelin levels, an orexigenic hormone, of SDT rats were significantly higher than those of SD rats at 38 weeks of age. Active ghrelin production and suppression of insulin or leptin may be concerned with diabetic hyperphagia [17]. In female rats as well, free fatty acids and TG were higher at 25 weeks of age before the onset of diabetes, compared with normal SD rats [13]. SDT rats fed high-sucrose diet showed dyslipidemia and insulin resistance; however, the incidence of hyperglycemia was suppressed. The milder degrees of pancreatic abnormalities in high-sucrose fed SDT rats may be considered as the reason [18].

### 3.4. Glucose Metabolism in the Liver

 As for the glucose metabolism-related enzymes in the liver, glucokinase mRNA level and glycogen content in the liver decreased in SDT rats at 16 weeks of age, suggesting that glucose metabolism in the liver is already abnormal before the onset of diabetes. After the onset of disease, mRNA expression of gluconeogenesis enzymes such as phosphoenolpyruvate carboxykinase (PEPCK), fructose-1,6-bisphosphatase (FBPase), and glucose-6-phosphatase (G6Pase) increased [15, 19].

### 3.5. Pancreatic Islets

**Figure 4:**
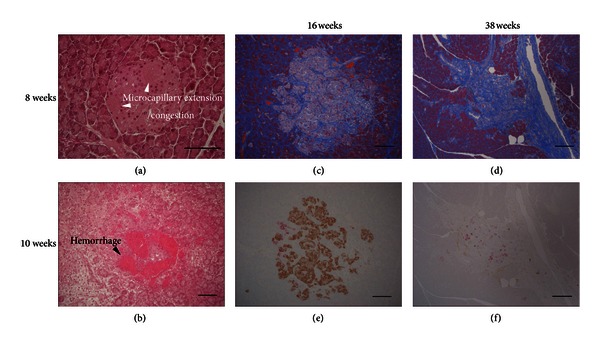
Histopathological observations in pancreas (islet) of SDT rats. Histopathological changes in the pancreatic islets of the male SDT rats. Congestion or microcapillary extension (a) and hemorrhage (b) in the pancreatic islets were observed (HE stain). Replacement of islet cells by connective tissues with advanced fibrosis (c, d) (MT stain). Immunohistochemistry of islet shows almost all of the *β*-cells (brown, insulin immunoreactive) disappeared from the islets, whereas *α*-cells (pink, glucagon immunoreactive) were still observed at 38 weeks (e, f). Bar = 100 *μ*m. Figures are modified from [8].

 In SDT rats, the number of pancreatic islets and the area of *β*-cells decreased with almost normal glucose tolerance at 10 weeks of age, compared with normal SD rats of the same age. Around 8 weeks, pancreatic islets with congestion and capillary dilation were sporadically found with those with hemorrhage and edema in the same sections (Figures 4(a) and 4(b)). Later, probably accompanying findings such as inflammation and fibrosis in or around the pancreatic islets extended, and fibrosis, hemosiderin deposition and marked decrease in *β*-cells were observed in almost all pancreatic islets at 20 weeks (Figures 4(c) and 4(d)). In SDT rats that developed diabetes, atrophy of pancreatic islets occupied by collagenous fibers and virtual disappearance of *β*-cells was observed (Figures 4(e) and 4(f)) [5, 6, 8]. These changes in pancreatic islets starting from hemorrhage were observed in female rats around the same time with those in males [13]. Higher sensitivity to streptozotocin (STZ), that has selective toxicity to pancreatic *β*-cells, is also suggesting a pancreatic weakness of SDT rats [9].

 During the course of the disappearance of *β*-cells, no lymphocyte infiltration was observed, unlike in type 1 diabetes (T1D) models such as nonobese diabetic (NOD) mice [20] or Biobreeding (BB) rats [21], but the concentration of interleukin-18 (IL-18), an inflammatory cytokine, transiently increased at 9 weeks, resulting in a corresponding increase in interferon gamma (IFN-*γ*) and nitric oxide (NO) production by spleen cells and peripheral leukocytes, respectively, as well as macrophage infiltration around the pancreatic islet tissue. In SDT rats, the number of white blood cells is increased at 8 weeks. It was immunohistologically found that the IL-18 receptor and inducible NO synthase (iNOS) were expressed in pancreatic islet cells. These findings indicate that the development of diabetes in SDT rats may be due to the damage of pancreatic islets resulting from a transient increase in the IL-18 concentration through direct effects on the cells and secondary effects via local macrophage infiltration [22].

 Pancreas transplantation is generally performed in patients with T1D, but exceptionally in those with T2D, improving insulin sensitivity in both cases. In SDT rats, it is suggested that the elimination of glucose toxicity following pancreas allotransplantation may induce the pancreatic expression of pancreatic and duodenal homeobox 1 (PDX-1), a homeodomain transcription factor, inhibiting the destruction of pancreatic islets and promoting the regeneration of pancreatic islets and *β*-cells [23, 24].

## 4. Analysis of Responsible Genes

 Development of diabetes in SDT rats is genetically regulated. Based on the results of genetic analyses using two control strains, seven quantitative trait loci (QTLs) involved in the impairment of glucose tolerance are currently mapped on the rat genome (Table 1) [25–27]. In a backcross experiment with Brown Norway (BN) rats, QTLs involved in the impairment of glucose tolerance in SDT rats were identified on chromosomes 1, 2, and X, which were named *Gisdt1*, *Gisdt2*, and *Gisdt3*, respectively. It is found that homozygosity or hemizygosity for the SDT allele in each of these QTLs markedly increases the risk of hyperglycemia (diabetes), and the interactions between the QTLs synergistically worsen glucose intolerance [26]. In an intercross experiment with F344 rats, furthermore, QTLs involved in the impairment of glucose tolerance in SDT rats were identified on chromosomes 3, 8, 13, and 14, which were named *Dmsdt1*, *Dmsdt2*, *Dmsdt3*, and *Dmsdt4*, respectively. To evaluate the effects of these QTLs on the development of diabetes, *χ* ^2^ test was performed using F2 rats with normal glucose tolerance and those with diabetes, showing that *Dmsdt1* is the most influential on the development of diabetes. Subsequently, congenic rats were generated by transferring *Dmsdt1* to F344 rats, and histological analysis was performed, revealing histopathological changes such as inflammation and fibrosis in the pancreas in the congenic rats. These results show that *Dmsdt1* is the major locus responsible for pancreatic lesions in SDT rats [25].

## 5. Complications

### 5.1. Ocular Complications

#### 5.1.1. Retinopathy

 Of many diabetic ocular complications, retinopathy, cataract, and neovascular glaucoma (hemorrhagic glaucoma) are the most important clinically. SDT rat is the first diabetic model with all of these complications [5, 6, 28–32]. 

**Figure 5:**
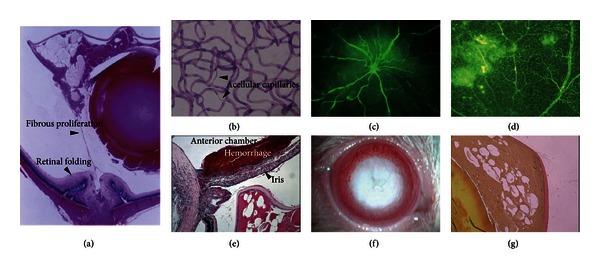
Diabetic ocular complications in SDT rats. Large retinal folds are seen in the midperipheral retina and around the optic disk. A tractional retinal detachment is observed with fibrous proliferation (a) (HE stain). Retinal trypsin digestion shows acellular capillaries (b) (HE stain). Tortuous vessels and extensive fluorescein leakages are observed in retinal flat mounts from SDT rats (c, d). Massive hemorrhage in the anterior chamber associated with proliferation around the iris is seen in some severe case (e) (HE stain). The mature cataract is observed clearly in the dilated pupil (f). The sclerotic nucleus floats in a liquefied lens cortex. Vacuolation, disintegration of the lens fibers, and Morgani's globules are observed in the lens cortex (g) (Elastica van Gieson stain). Figures are modified from [6, 28–31].

 Further progression of the disease was characterized by proliferative retinopathy, with tractional retinal detachment primarily in the optic disc due to fibrovascular membrane resulting from retinal neovascular vessels (Figure 5(a)) [5, 6, 28–32]. The vascular pathological examination by trypsin digestion method showed a few capillary aneurysms, but revealed capillary narrowing and pericyte loss in SDT rats (Figure 5(b)). In fluorescein angiography, abnormal retinal vasodilatation was observed in some animals and may correspond to venous beading in human retinopathy [5, 28–32]. In addition, severe fluorescein leakage almost corresponding to the area affected by tractional retinal detachment [28–32] (Figures 5(c) and 5(d)) was revealed. The prevalence of diabetic retinopathy was 8% at 35 to 50 weeks of age, but increased to approximately 80% at 51 to 60 weeks and 100% at 61 to 82 weeks [29].

 At 44 weeks, electroretinogram (ERG) revealed the delay and reduction of oscillatory potentials (OPs) and a- and b-waves [31, 33], as is the case with human diabetic retinopathy.

 It is known that not only microangiopathy, but also neurodegeneration occurs in the human diabetic retina. In comparison of changes in the death of neuroretinal cells as well as expression of glial fibrillary acidic protein (GFAP: a marker protein for glial cells) and water channel aquaporins (AQPs) over time in SDT rats, the AQP expression profile in astrocytes in the nerve fiber layer shifted from AQP-4 to AQP-1 in the retinas of SDT rats at 40 weeks, when the apoptosis of retinal ganglion cells (RGCs) was accelerated. AQP-0 was predominantly expressed in the bipolar cells of the nondiabetic rat, whereas it was also expressed in the retinal nerve fibers of diabetic rat. The disrupted water transport between astrocytes and retinal nerve fibers may be associated with the apoptosis of RGC induced by diabetes [34, 35].

 In SDT rats, the angiotensin II (AII) concentration in circulating blood was low at 15, 30, and 45 weeks, but the angiotensin-converting enzyme (ACE) activity specifically increased in the eye without change in the aortic ACE activity at 45 weeks. In addition, continuous treatment with AII resulted in increased retinal expression of the vascular endothelial growth factor (VEGF) gene. These findings suggest that specifically increased AII formation in the eye may play an important role in retinal VEGF expression in SDT rats [36]. Furthermore, advanced glycation end products (AGEs) such as carboxymethyllysine (CML) were expressed with VEGF in the retina and may be involved in retinopathy in SDT rats [37]. On the other hand, angiogenesis was observed with VEGF expression, but it has been reported that retinal neovascularization is not associated with retinal nonperfusion in SDT rats, unlike human diabetic retinopathy [38]. Unlike human diabetic retinopathy, the retinal capillary bed is hardly obstructed in SDT rats, indicating that increased expression of the pigment epithelium-derived factor (PEDF) results in the suppression of diabetic retinal vascular disorder and less obstruction of the retinal capillary bed in SDT rats [39]. 

#### 5.1.2. Neovascular Glaucoma

 In human retinopathy, severely advanced retinal ischemia is finally associated with angiogenesis in the iris and anterior chamber angle, presenting with neovascular glaucoma. In SDT rats as well, advanced retinopathy is associated with fibrovascular membrane around the iris and sometimes with anterior chamber hemorrhage. These pathological conditions in SDT rats indicate the iris neovascularization (rubeosis). Neovascular membrane around the pupil may cause posterior synechiae and might develop neovascular glaucoma finally. SDT rat is a first model of iris neovascularization and consequent neovascular glaucoma [5, 6, 28, 29] (Figure 5(e)).

#### 5.1.3. Cataract

 In male SDT rats, the prevalence of cataract is virtually 100% at 40 weeks of age. Starting with opacity at the posterior pole of the lens, the findings of mature cataract are finally observed (Figures 5(f) and 5(g)). Nuclear sclerosis progresses, and the cortex is highly opacified. Pathological findings include swollen lens fibers, liquefaction, vacuolation, abnormal configuration, and formation of Morgagnian droplets as well as partial proliferation of fibroblastoid cells. Advanced cataract is associated with capsular rupture, probably related to swollen lens [5, 6, 28–32]. 

 These ocular complications in SDT rats have been shown to be prevented by normalizing blood glucose with insulin treatment or pancreas transplantation and demonstrated to result from the long-term exposure to high blood glucose [31, 40]. Corneal disorder, optic neuropathy, and uveitis are also known as ocular complications in T2D. Though uveitis is not observed in SDT rats, corneal disorder and optic neuropathy are not well investigated.

### 5.2. Nephropathy

**Figure 6:**
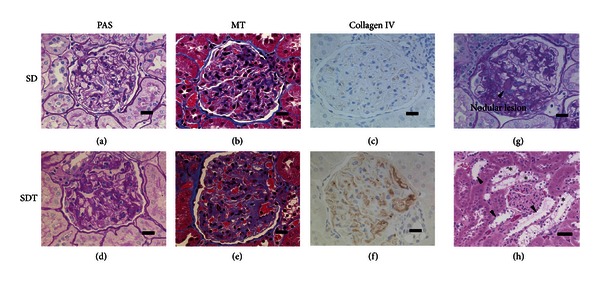
Histopathological observations in kidney of SDT rats. Histological and immunohistological analysis of glomeruli in SD rats (a–c) and SDT rats (d–f). In the glomeruli of SDT rats, basement membrane thickening and mesangial matrix proliferation were observed at 50 weeks of age (bar = 20 *μ*m). Nodular lesions were found in a few glomeruli from 68-week-old SDT rats (g) (PAS stain, bar = 20 *μ*m). In the renal tubules of SDT rats, dilation (arrowhead) and glycogen deposition in the epithelium (∗) were observed (h) (HE stain, bar = 50 *μ*m). Figures are modified from [41].

In SDT rats, renal lesions appeared at 24 weeks of age, including the thickening of the glomerular loop and glycogen deposition in the tubular epithelium (Armanni-Ebstein lesion), dilatation of the renal tubule lumen, and increased hyaline casts. As for the glomerular lesions, slight thickening of the loop was apparent at 24 weeks and consistent with mesangial proliferation as shown by PAS, Masson's trichrome stain, and type IV collagen immunostaining (Figures 6(a)–6(f)). Mesangial proliferation intensified with age, and nodular lesions (Kimmelstiel-Wilson-like nodules) suggestive of more severe glomerular lesions were slightly observed at 68 weeks (Figure 6(g)). On the other hand, the renal tubular lesions markedly increased with age, with a severe increase in tubular glycogen deposition at 50 and 68 weeks (Figure 6(h)). In addition, urine volume, urine protein, and urine albumin increased with blood glucose at 24 weeks and thereafter, and these changes may be consistent with the development and progression of renal lesions [41, 42]. These renal lesions were also improved by blood glucose control with insulin and thus shown to result from the exposure to high blood glucose [41, 42].

 In a study evaluating the involvement of oxidative stress and NO in the mechanism for the progression of diabetic nephropathy in SDT rats, the blood asymmetric dimethylarginine (ADMA) concentration and urinary excretion of oxidative stress markers 8-hydroxydeoxyguanosine (8-OHdG) and nitrogen oxide (NOx) increased in SDT rats at 36 weeks, compared with insulin-treated SDT rats and normal SD rats. In addition, renal tissue analysis revealed glomerular hypertrophy and mesangial proliferation, and immunostaining analysis showed that the glomerular 8-OHdG, endothelial NO synthase (eNOS), and nitrotyrosine scores increased. In SDT rats, eNOS and NO increased despite the increase in ADMA and may thus play an important role in the progression of diabetic nephropathy together with oxidative stress [43]. Metformin, an AMP-activated kinase (AMPK) activator, decreased renal 8-OHdG levels and subsequent podocyte loss, in spite of the limited effects on hyperglycemia [44].

### 5.3. Neuropathy

#### 5.3.1. Peripheral Neuropathy

**Figure 7:**
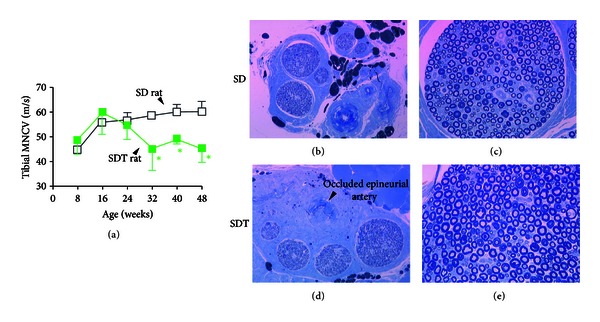
Diabetic peripheral neuropathy and histopathology in sural nerves of SDT rats. Serial changes of tibial motor nerve conduction velocity (MNCV) in male SD rats (white square) and male SDT rats (green square) (a). MNCV reduced after the onset of diabetes. Data represent means ± SD (*N* = 6). *P < 0.05 (versus age-matched SD rats, unpaired *t*-test). Morphologically, SDT rats revealed significant atrophy in myelinated nerve at 48 weeks of age. Occluded/thickened epineurial arterioles were found in SDT rats. Typical low (b, d) and high (c, e) magnification of microphotographs of sural nerves from SD rat and SDT rat (toluidine blue stain). Figures are modified from [45].

 Both motor nerve and sensory nerve are impaired under in diabetes. In an electrophysiological and morphological study of diabetic peripheral neuropathy (DPN) in SDT rats, the motor nerve conduction velocity (MNCV) was not different from that in normal SD rats until 6 months of age, but gradually decreased thereafter to 82% and 76% of that in normal SD rats at 10 and 12 months, respectively (Figure 7(a)) [45]. Sensory nerve conduction velocity (SNCV) is also decreased. Increased nerve sorbitol and fructose contents and decreased *myo*-inositol contents in SDT rats indicate that the polyol pathway is prominently involved in DPN. Ranirestat, an aldose reductase inhibitor (ARI) decreased sciatic nerve sorbitol levels and improved impaired sciatic MNCV [46].

 In the sural nerve cross-section, no neurologic deficit was observed, but degenerated nerves increased in SDT rats. In morphometry, the myelinated nerve area was not clearly different between the two groups at 6 months, but decreased in SDT rats at 12 months compared with normal SD rats. The number of blood vessels in the nerve sheath was not clearly different; however, occluded/thickened epineurial arterioles were found in SDT rats (Figures 7(b)–7(e)) [45, 47]. The increased intima possibly results decrease of nerve perfusion and may contribute to development of DPN in SDT rats. In summary, it is shown that SDT rats develop peripheral neuropathy associated with T2D after the onset of disease, including functional/morphological abnormalities of peripheral nerves and vascular lesions. 

#### 5.3.2. Autonomic Neuropathy

 Autonomic nerve is part of the peripheral nervous system and transmits impulses from the central nervous system to peripheral organ systems. In diabetes, autonomic nerve is also known to be impaired. Symptoms probably due to diabetic diarrhea are observed in SDT rats. In charcoal propulsion test, gastrointestinal motility increased in SDT rats with higher fecal water content at 28 weeks of age compared with insulin-treated SDT rats and normal SD rats [48]. In addition, jejunum and ileum weights and mucosal weight increased, and the lumen diameter and villous height were longer indicating that more nutrients are absorbed with longer villi in diabetes [15, 16, 48].

 In a study of voiding dysfunction in SDT rats, voiding pressure, voided volume per micturition, and intermicturition interval tended to increase from 22 weeks to 36 weeks of age compared with normal rats. SDT rats may have chronic diabetic dysuria, which progresses with age [49]. 

### 5.4. Other Complications

**Figure 8:**
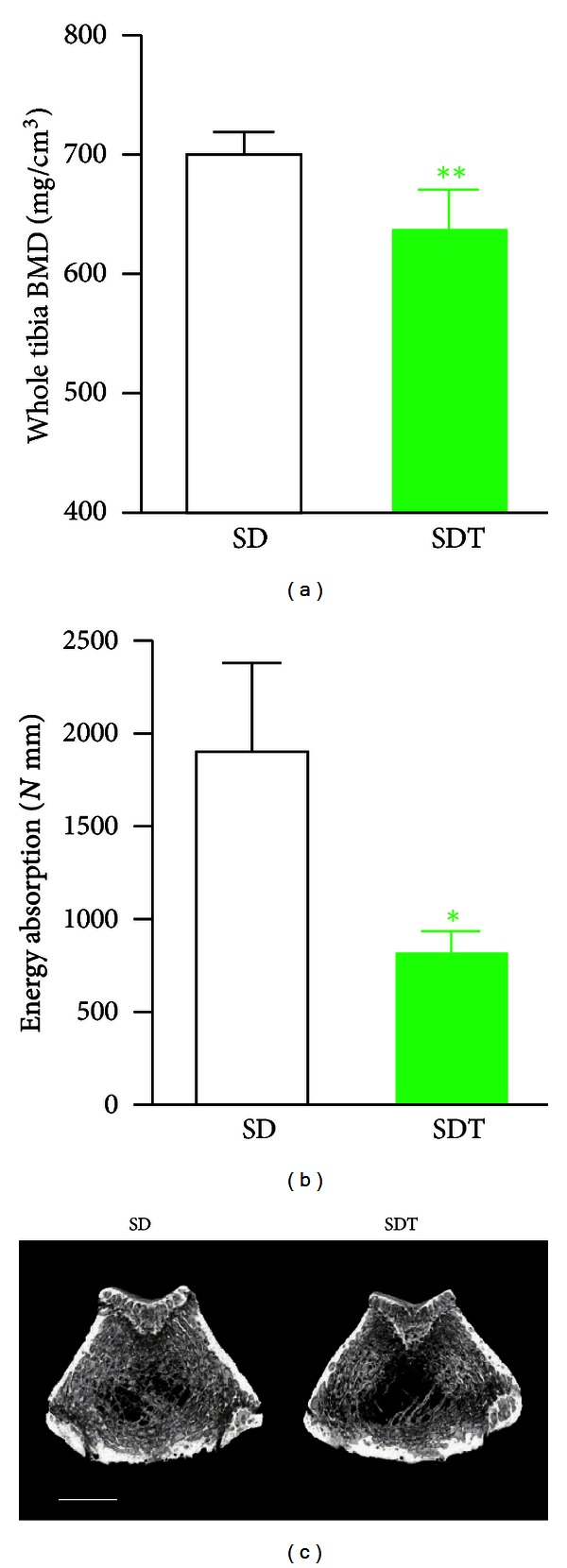
Bone properties of SDT rats. Bone mineral density (BMD) of the whole tibia (a) and mechanical property of the femur midshaft (b) are clearly indicating diabetes-induced osteoporosis in SDT rats. White column: male SD rats, green column: male SDT rats (40 weeks of age). Data represent means ± SD (*N* = 5). **P < 0.01, *P < 0.05 (versus SD rats, unpaired *t*-test). The reconstructed bone microstructure 3D images of femur from 40 weeks SDT rats show the obvious bone loss (c) (bar = 2 mm). Figures are modified from [50].

 Patients with T2D also suffer other complications than the microvascular complications mentioned above. Immunodeficiency, delayed wound healing, skin ulcer, and osteoporosis are well known. Among these complications, osteoporosis has been reported in SDT rat. In a study of bone lesions in SDT rats with focus on bone density and bone morphometry, bone formation and resorption decreased in SDT rats at 36 weeks of age compared with normal SD rats, but improved in insulin-treated SDT rats. Bone density and strength also decreased in SDT rats compared with normal SD rats (Figure 8). Bone lesions in SDT rats were characterized by decreased bone density and low-turnover bone lesions, as seen with T2D primarily due to decreased insulin secretion, and improved with insulin treatment, indicating the deep involvement of diabetic pathology [50, 51]. The study treating carvedilol, a *β* blocker possessing an antioxidant effect, is also suggesting the involvement of oxidative stress on this low-turnover bone disease in SDT rats [52]. 

## 6. Application to Treatment

 Use of animal models is essential to the development of diabetic drugs. Currently, SDT rats are used for the development and application of several diabetic drugs. In addition to insulin [31, 41], sulfonylurea (tolbutamide) and DPP IV inhibitor (JTP-76209) [12], *α*-glucosidase inhibitor (voglibose) [53], SGLT inhibitor (phlorizin) [15, 54], and perilla (shiso) tea [55] lowered the blood glucose level of SDT rats. 

 It has been reported that diabetic microangiopathy is caused by increased tissue protein kinase C-beta (PKC-*β*) activity at high blood glucose levels. In SDT rats at 32 weeks of age, abnormal retinal function such as delayed OPs in ERG were observed. In addition, peripheral and autonomic neuropathies such as decreased caudal MNCV, electrocardiographic coefficient of variation of *R-R* interval (CV_*R*-*R*_), and thermal hypoalgesia were observed. These diabetic complications were improved after 12-week treatment with a PKC-*β* inhibitor JTT-010. However, histopathological changes including retinal thickening primarily in the optic disc at 68 weeks of age were not improved. Since the tissue PKC activity increased after the onset of diabetes in SDT rats, JTT-010 may have suppressed diabetic neuropathy by inhibiting the PKC-*β* activity. However, the retinal histopathological findings were not affected in SDT rats that developed diabetes along earlier, indicating that factors other than PKC-*β* activation are deeply involved in the progression of ocular complications in SDT rats [56]. Benfotiamine, a transketolase activator that reduces major pathways involved in diabetic microvascular complications (polyol pathway, hexosamine pathway, AGE pathway, and diacylglycerol-protein kinase C (DAG-PKC) pathway) also exhibits effects on peripheral nerve function in SDT rats [45].

 In a large-scale clinical study, it was reported that candesartan, an AII type 1 receptor blocker (ARB), inhibited the progression of retinopathy in type 2 diabetic patients [57]. In an efficacy study of an telmisartan for the progression of ocular lesions in SDT rats, the blood glucose level was not changed, but blood pressure was decreased by telmisartan. Under these conditions, delayed OPs and a-wave in ERG were prevented by telmisartan. In fluorescein fundus angiography, fluorescein leakage in SDT rats was decreased by telmisartan, suggesting that the ARB may inhibit the development of proliferative retinopathy in SDT rats [58]. It has also been reported that ARBs (candesartan and olmesartan) improved coronary angiogenesis, cardiomyocyte fibrosis, and hypertrophy associated with the progression of diabetes in SDT rats [59, 60]. In addition, candesartan decreased the pentosidine, a biomarker for AGE, content in the lens/vitreous body in SDT rats at 44 weeks of age, and immunohistologically, it inhibited the accumulation of pentosidine in the retinal vascular wall and decreased retinal VEGF mRNA expression [61]. These findings indicate that ARBs can inhibit the development of proliferative diabetic retinopathy by inhibiting AGE formation. Furthermore, it has been reported that cataract and retinopathy in SDT rats were prevented by ARI fidarestat [62] and ranirestat [46], AGE inhibitor aminoguanidine [63], and *α*1/*β* blocker nipradilol [64].

 With application of gene therapy, the soluble VEGF receptor (sFlt-1) gene was introduced into the retina in SDT rats to evaluate the preventive effect of sFlt-1 expressed in the retina against diabetic retinopathy. At 57 weeks of age, fluorescein fundus angiography revealed that the development of retinopathy was inhibited in the retina engineered to express sFlt-1 using an adeno-associated virus (AAV) vector as compared with the contralateral naïve retina. Since the local introduction of sFlt-1 gene in the retina with the use of an AAV vector is effective in preventing the development of retinopathy in SDT rats, gene therapy for diabetic retinopathy with antiangiogenic factors will be a promising therapeutic option for human patients [65, 66]. 

## 7. Conclusion

 Newly established SDT rats show ocular complications similar to those in human diabetes. Proliferative retinopathy, especially, resulting from retinal neovascular vessels is a unique characteristic of this model. No other models show such histology in eyes. In addition, diabetic neuropathy (e.g., mesangial proliferation, nodular lesion) and diabetic peripheral/autonomic nephropathy (e.g., decreased NCVs, hypoalgesia, diarrhea, and voiding dysfunction) seem to be caused by postprandial long-term hyperglycemia in SDT rats. Bone disorder such as decreased bone density and low-turnover bone lesions are also observed. At younger age, glucose intolerance, caused by pancreatic islet lesions with inflammatory cell infiltration and fibroblasts, is also a characteristic of this animal. Taking advantage of these features, SDT rat has been used for evaluating antidiabetic drugs and drugs/gene therapy for diabetic complications. Although there are insurmountable discrepancies between human and rodents, SDT rat seems to be a better animal model of diabetes than other models.

 In conclusion, findings indicate that the SDT rats should be a potential T2D model for studies on the pathogenesis and treatment of diabetes and its complications.

## Figures and Tables

**Table 1:** QTLs involved in glucose intolerance in SDT rats.

QTL	Chr.	Position^a^	Trait^b^	Inheritance mode	Cross
*Gisdt1 *	1	*D1Mit3 *	Postprandial	Recessive	(BN × SDT) × SDT
*Gisdt2 *	2	*D2Got147 *	Postprandial	Recessive	(BN × SDT) × SDT
*Gisdt3 *	X	*DXWox20 *	Postprandial	X-linked	(BN × SDT) × SDT
*Dmsdt1 *	3	*D3Mit12 *	Postprandial	Dominant, additive	(F344 × SDT) F2
*Dmsdt2 *	8	*D8Rat46 *	Fasting, Postprandial	Recessive	(F344 × SDT) F2
*Dmsdt3 *	13	*D13Arb5 *	Fasting	Recessive	(F344 × SDT) F2
*Dmsdt4 *	14	*D14Arb18 *	Postprandial	Additive	(F344 × SDT) F2

^a^The SSLP markers which are the closest to maximum peaks of QTLs are shown.

^b^Postprandial: postprandial blood glucose levels, fasting: fasting blood glucose levels.

Seven highly significant QTLs (*Gisdt1*, *Gisdt2*, *Gisdt3*, *Dmsdt1*, *Dmsdt2*, *Dmsdt3*, and *Dmsdt4*) for glucose intolerance have been identified in SDT rats. The table is summarized from [25–27].

## References

[1] Wild S, Roglic G, Green A, Sicree R, King H (2004). Global prevalence of diabetes: estimates for the year 2000 and projections for 2030. *Diabetes Care*.

[2] Zimmet P, Alberti KGMM, Shaw J (2001). Global and societal implications of the diabetes epidemic. *Nature*.

[3] International Diabetes Federation (2011). *IDF Diabetes Atlas*.

[4] Shinohara M (2011). Establishment and clinical features in spontaneously diabetic torii rat. *Open Diabetes Journal*.

[5] Shinohara M, Masuyama T, Kakehashi A, Shafrir E (2007). The Spontaneously Diabetic Torii (SDT) rat with retinopathy lesions resembling those of humans. *Animal Models of Diabetes: Frontiers in Research*.

[6] Shinohara M, Masuyama T, Shoda T (2000). A new spontaneously diabetic non-obese torii rat strain with severe ocular complications. *International Journal of Experimental Diabetes Research*.

[7] Shinohara M, Oikawa T, Sato K, Kanazawa M (2011). Effect of oophorectomy and estrogen administration on diabetic pathogenesis in female Spontaneously Diabetic Torii rats. *Open Diabetes Journal*.

[8] Masuyama T, Komeda K, Hara A (2004). Chronological characterization of diabetes development in male Spontaneously Diabetic Torii rats. *Biochemical and Biophysical Research Communications*.

[9] Ohta T, Miyajima K, Yamada T (2011). Pathophysiological changes in pre-diabetic stage of Spontaneously Diabetic Torii (SDT) rats. *Journal of Animal and Veterinary Advances*.

[10] Masuyama T (2011). Characteristics of diabetes in the SDT rat. *Open Diabetes Journal*.

[11] Ohta T, Shinohara M, Yamamoto T, Yamada T (2012). Pancreatic abnormalities at a young age in Spontaneously Diabetic Torii (SDT) rats. *Journal of Animal and Veterinary Advances*.

[12] Matsui K, Oda T, Nishizawa E (2009). Pancreatic function of spontaneously diabetic torii rats in pre-diabetic stage. *Experimental Animals*.

[13] Shinohara M, Oikawa T, Sato K, Kanazawa Y (2004). Glucose intolerance and hyperlipidemia prior to diabetes onset in female Spontaneously Diabetic Torii (SDT) rats. *Experimental Diabesity Research*.

[14] Ishii Y, Ohta T, Sasase T (2010). A high-fat diet inhibits the progression of diabetes mellitus in type 2 diabetic rats. *Nutrition Research*.

[15] Mera Y, Morinaga H, Ohta T, Sasase T (2011). Glucose and lipid metabolism in Spontaneously Diabetic Torii rat. *Open Diabetes Journal*.

[16] Sasase T, Morinaga H, Yamamoto H (2007). Increased fat absorption and impaired fat clearance cause postprandial hypertriglyceridemia in Spontaneously Diabetic Torii rat. *Diabetes Research and Clinical Practice*.

[17] Mifune H, Nishi Y, Tajiri Y (2012). Increased production of active ghrelin is relevant to hyperphagia in nonobese Spontaneously Diabetic Torii rats. *Metabolism*.

[18] Ohta T, Miyajima K, Yamada T (2010). Changes in glycolipid metabolism during a high-sucrose feeding in Spontaneously Diabetic Torii (SDT) rats, a genetic model of nonobese type 2 diabetes. *Journal of Animal and Veterinary Advances*.

[19] Morinaga H, Yamamoto H, Sakata K (2008). Characterization of hepatic glucose metabolism disorder with the progress of diabetes in male Spontaneously Diabetic Torii rats. *Journal of Veterinary Medical Science*.

[20] Makino S, Harada M, Kishimoto Y, Hayashi Y (1986). Absence of insulitis and overt diabetes in athymic nude mice with NOD genetic background. *Jikken dobutsu. Experimental animals*.

[21] Greiner DL, Handler ES, Nakano K (1986). Absence of the RT-6 T cell subset in diabetes-prone BB/W rats. *Journal of Immunology*.

[22] Inokuchi C, Ueda H, Hamaguchi T (2009). Role of macrophages in the development of pancreatic islet injury in spontaneously diabetic torii rats. *Experimental Animals*.

[23] Shimada K, Ito T, Miao G (2008). Regeneration of *β* Cells in the Native Pancreata After Syngeneic and Allogeneic Pancreas Transplantations in Spontaneously Type 2 Diabetic Torii Rats. *Transplantation Proceedings*.

[24] Shimada K, Ito T, Tanemura M (2008). Development of *β*-Cells in the Native Pancreas After Pancreas Allo-Transplantation in the Spontaneously Diabetic Torii Rat. *Journal of Surgical Research*.

[25] Fuse M, Yokoi N, Shinohara M (2008). Identification of a major locus for islet inflammation and fibrosis in the spontaneously diabetic Torii rat. *Physiological Genomics*.

[26] Masuyama T, Fuse M, Yokoi N (2003). Genetic analysis for diabetes in a new rat model of nonobese type 2 diabetes, Spontaneously Diabetic Torii rat. *Biochemical and Biophysical Research Communications*.

[27] Yokoi N, Fuse M, Seino S (2011). Genetics of the Spontaneously Diabetic Torii rat. *Open Diabetes Journal*.

[28] Kakehashi A (2011). Diabetic ocular complications in the SDT rat. *Open Diabetes Journal*.

[29] Kakehashi A, Saito Y, Mori K (2006). Characteristics of diabetic retinopathy in SDT rats. *Diabetes/Metabolism Research and Reviews*.

[30] Sasase T (2010). Pathophysiological characteristics of diabetic ocular complications in Spontaneously Diabetic Torii rat. *Journal of Ophthalmology*.

[31] Sasase T, Ohta T, Ogawa N (2006). Preventive effects of glycaemic control on ocular complications of Spontaneously Diabetic Torii rat. *Diabetes, Obesity and Metabolism*.

[32] Shoda T, Shinohara M, Takahashi T, Miyajima K, Kakehashi A, Miyakawa Y (2007). Histopathological features of diabetic ocular complications in the Spontaneously Diabetic Torii (SDT) rat. *Journal of Toxicologic Pathology*.

[33] Okuno T, Oku H, Sugiyama T, Ikeda T (2008). Electroretinographic study of spontaneously diabetic Torii rats. *Documenta Ophthalmologica*.

[34] Fukuda M, Naka M, Mizokami J, Negi A, Nakamura M (2011). Diabetes induces expression of aquaporin-0 in the retinal nerve fibers of spontaneously diabetic Torii rats. *Experimental Eye Research*.

[35] Fukuda M, Nakanishi Y, Fuse M (2010). Altered expression of aquaporins 1 and 4 coincides with neurodegenerative events in retinas of spontaneously diabetic Torii rats. *Experimental Eye Research*.

[36] Fukumoto M, Takai S, Ishizaki E (2008). Involvement of angiotensin II-dependent vascular endothelial growth factor gene expression via NADPH oxidase in the retina in a type 2 diabetic rat model. *Current Eye Research*.

[37] Toyoda F, Kakehashi A, Hashimoto K (2011). Accumulation of AGEs and VEGF in eyes of SDT rats. *Open Diabetes Journal*.

[38] Yamada H, Yamada E, Higuchi A, Matsumura M (2005). Retinal neovascularisation without ischaemia in the spontaneously diabetic Torii rat. *Diabetologia*.

[39] Matsuoka M, Ogata N, Minamino K, Higuchi A, Matsumura M (2006). High levels of pigment epithelium-derived factor in the retina of a rat model of type 2 diabetes. *Experimental Eye Research*.

[40] Miao G, Ito T, Uchikoshi F (2004). Stage-dependent effect of pancreatic transplantation on diabetic ocular complications in the Spontaneously Diabetic Torii rat. *Transplantation*.

[41] Ohta T, Matsui K, Miyajima K (2007). Effect of insulin therapy or renal changes in spontaneously diabetic Torii rats. *Experimental Animals*.

[42] Ohta T, Sasase T (2011). Diabetic nephropathy in Spontaneously Diabetic Torii (SDT) rats. *Open Diabetes Journal*.

[43] Fujii H, Kono K, Nakai K (2010). Oxidative and nitrosative stress and progression of diabetic nephropathy in type 2 diabetes. *American Journal of Nephrology*.

[44] Kim J, Shon E, Kim CS, Kim JS (2012). Renal podocyte injury in a rat model of type 2 diabetes is prevented by metformin. *Experimental Diabetes Research*.

[45] Sasase T, Ohta T (2011). Diabetic neuropathy in Spontaneously Diabetic Torii rat. *Open Diabetes Journal*.

[46] Ota A, Kakehashi A, Toyoda F (2013). Effects of long-term treatment with ranirestat, a potent aldose reductase inhibitor, on diabetic cataract and neuropathy in Spontaneously Diabetic Torii rats. *Experimental Diabetes Research*.

[47] Yamaguchi T, Sasase T, Mera Y (2012). Diabetic peripheral neuropathy in Spontaneously Diabetic Torii-Leprfa (SDT Fatty) rats. *Journal of Veterinary Medical Science*.

[48] Yamada K, Hosokawa M, Fujimoto S (2007). The spontaneously diabetic Torii rat with gastroenteropathy. *Diabetes Research and Clinical Practice*.

[49] Matsumoto Y, Torimoto K, Matsuyoshi H (2009). Long-term effects of diabetes mellitus on voiding function in a new model of type 2 diabetes mellitus, the Spontaneously Diabetic Torii (SDT) rat. *Biomedical Research*.

[50] Kimura S, Sasase T, Ohta T, Sato E, Matsushita M (2012). Characteristics of bone turnover, bone mass and bone strength in Spontaneously Diabetic Torii-Leprfa rats. *Journal of Bone and Mineral Metabolism*.

[51] Fujii H, Hamada Y, Fukagawa M (2008). Bone formation in spontaneously diabetic Torii-newly established model of non-obese type 2 diabetes rats. *Bone*.

[52] Goto S, Fujii H, Kono K (2011). Carvedilol ameliorates low-turnover bone disease in non-obese type 2 diabetes. *American Journal of Nephrology*.

[53] Ohta T, Miyajima K, Shinohara M, Yamamoto T, Yamada T (2011). Inhibition of postprandial hyperglycemia prevents the incidence of diabetes in Spontaneously Diabetic Torii (SDT) rats. *Journal of Animal and Veterinary Advances*.

[54] Ohta T, Morinaga H, Yamamoto T, Yamada T (2012). Effect of phlorizin on metabolic abnormalities in Spontaneously Diabetic Torii (SDT) rats. *Open Journal of Animal Sciences*.

[55] Kishi H, Komatsu W, Miura Y, Kawanobe T, Nonaka T, Ohhira S (2010). Effects of habitual perilla (shiso) tea drinking on the incidence of diabetes mellitus in spontaneously diabetic trii (SDT) rats. *Bioscience, Biotechnology and Biochemistry*.

[56] Sasase T, Morinaga H, Abe T (2009). Protein kinase c beta inhibitor prevents diabetic peripheral neuropathy, but not histopathological abnormalities of retina in Spontaneously Diabetic Torii rat. *Diabetes, Obesity and Metabolism*.

[57] Sjølie AK, Klein R, Porta M (2008). Effect of candesartan on progression and regression of retinopathy in type 2 diabetes (DIRECT-Protect 2): a randomised placebo-controlled trial. *The Lancet*.

[58] Hasegawa G, Fukui M, Hosoda H (2009). Telmisartan, an angiotensin II type 1 receptor blocker, prevents the development of diabetes in male Spontaneously Diabetic Torii rats. *European Journal of Pharmacology*.

[59] Jin D, Takai S, Sugiyama T (2009). Long-term angiotensin II blockade may improve not only hyperglycemia but also age-associated cardiac fibrosis. *Journal of Pharmacological Sciences*.

[60] Matsuda T, Muto S, Fujisawa G (2012). Heart angiotensin II-induced cardiomyocyte hypertrophy suppresses coronary angiogenesis and progresses diabetic cardiomyopathy. *American Journal of Physiology*.

[61] Sugiyama T, Okuno T, Fukuhara M (2007). Angiotensin II receptor blocker inhibits abnormal accumulation of advanced glycation end products and retinal damage in a rat model of type 2 diabetes. *Experimental Eye Research*.

[62] Kakehashi A, Takezawa M, Toyoda F (2011). Aldose reductase inhibitor fidarestat prevents diabetic ocular complications in Spontaneously Diabetic Torii rats. *Open Diabetes Journal*.

[63] Toyoda F, Kakehashi A, Ota A (2011). Prevention of proliferative diabetic retinopathy and cataract in SDT rats with aminoguanidine, an anti-advanced glycation end product agent. *Open Diabetes Journal*.

[64] Kinoshita N, Kakehashi A, Dobashi Y (2011). Effects of topical nipradilol on early diabetic retinopathy in SDT rats. *Open Diabetes Journal*.

[65] Ideno J, Mizukami H, Kakehashi A (2007). Prevention of diabetic retinopathy by intraocular soluble flt-1 gene transfer in a spontaneously diabetic rat model. *International Journal of Molecular Medicine*.

[66] Mizukami H, Urabe M, Kume A, Ozawa K (2011). Gene therapy for diabetic retinopathy in animal models and humans. *Open Diabetes Journal*.

